# Laparoscopic liver resections

**DOI:** 10.4103/0972-9941.18993

**Published:** 2005-09

**Authors:** Alfred Cuschieri

**Affiliations:** Professor of Surgery, Scuola Superiore di Studi Universitari, Pisa, Italy

**Keywords:** hepatic tumour, laparoscopy, liver resection

## Abstract

Though still practiced in only a few centres worldwide, laparoscopic liver resections, particularly left hepatectomy offer advantages over the conventional open approach in two important respects: reduced operative blood loss and lower major postoperative morbidity. Two approaches are used: the totally laparoscopic and the hand-assisted technique, which in the author's opinion facilitates both the execution and safety of these procedures, especially major resection of the right liver (right hepatectomy and pluri-segmentectomies). Technologies, which have enabled hepatic resections include: laparoscopic contact ultrasound, linear cutting staplers, ultrasonic dissection, LigaSure and TissueLink. The components operative steps necessary for these resections as practised by the author are described in this review.

Although laparoscopic staging for intra-abdominal cancer including primary and secondary hepatic tumours has been in established practice for many years, laparoscopic liver resections are still in the early clinical evaluation stage and largely confined to major centres with expertise in hepato-biliary-pancreatic surgery. Nonetheless, the results to date have been uniformly favourable especially for left lobectomy and pluri-segmentectomies although right hepatectomy has been performed by the laparoscopically assisted or the hand-assisted laparoscopic surgical (HALS) approach.[1–18] The benefits commonly reported thus far include reduced blood loss, smoother postoperative period in patients with liver disease and a shorter postoperative hospital stay. Laparoscopic left lateral lobectomy (bi-segmentectomy 2–3) for tumours can be performed with relative ease and safety. In a recent case-control study,[14] 60 such laparoscopic resections using ultrasonic dissection, linear staplers, and portal pedicle clamping (when necessary) were compared with patients who underwent open left lateral lobectomies matched and selected from a liver resection database of open resections. Both groups were similar (age, type and size of the tumour presence of underlying liver disease). The findings of this study confirmed that compared to equivalent open hepatic resections, laparoscopic left lobectomies incurred a longer surgical time and longer portal triad clamping (39 *vs* 23 min), but were associated with decreased blood loss. There were no deaths in either group; and the morbidity rates were comparable (11% in the laparoscopic and 15% in the open group). There were no major postoperative complications of hepatic resection (haemorrhage, subphrenic collection or biliary leak) after laparoscopic resections, but some were observed in the open hepatic resections.

The HALS approach by facilitating these dissections and greatly increasing the safety (particularly in the ability for immediate control of major bleeding) will make quite a big difference to the uptake amongst hepato-biliary and general surgeons with an interest in liver surgery.[15–18]

The procedures, which are in established practice by the laparoscopic and HALS approach are:

- Extended laparoscopic staging.
- Hepatic resections.
- Laparoscopic *in situ* thermal ablation.
- Laparoscopic cryosurgery.
- Radical de-roofing of simple hepatic cysts.
- Hepatic surgery for parasitic cysts.

Whatever the procedure and irrespective of the approach, the safe execution demands (i) equivalent experience in open hepato-biliary surgery, (ii) expert knowledge of the surgical anatomy of the liver, (iii) familiarization with the safe use of ablative technologies and other energized equipment and (iv) good experienced operating team in a dedicated operating room. This review focuses on laparoscopic staging of hepatic tumours and o hepatic resections based on past experience of the author and on a literature review. The emphasis throughout the review is on practical operative issues relevant to these procedures.

## Laparoscopic staging of tumours

There is some controversy as to the necessity and value of routine simple visual inspection laparoscopy in the staging of certain intra-abdominal cancers. Undoubtedly, laparoscopy will detect seedling metastases and small hepatic deposits missed by preoperative thin slice multi detector CT or MRI. Some surgeons add lavage cytology to diagnostic laparoscopic visual inspection. This detects exfoliated tumours cells in pancreatic, gastrointestinal and ovarian cancers. The detection of these malignant exfoliated cells by immunohistochemical staining carries a poor prognosis but does not equate to inoperability. In essence, a positive lavage cytology (in the absence of overt detectable disseminated disease) does not dictate immediate surgical management.

The ability of visual inspection laparoscopy to assess resectability (as opposed to inoperability) remains relatively low.[19] It can be improved by *extended laparoscopy* combined with laparoscopic contact ultrasonography. [20–22] The technique of extended laparoscopy consists of full inspection of the peritoneal cavity, liver (with contact laparoscopic ultrasound scanning), entry and inspection of lesser sac, examination of porta hepatis, duodenum, transverse mesocolon, and coeliac and portal vessels. This procedure thus entails extensive dissection and is used to assess operability in patients with pancreatic cancer, hepatic neoplasms and gastroesophageal cancers where it often entails lymph node sampling. The value of extended laparoscopic was confirmed in a large study from the Memorial Sloane Kettering Hospital on 115 patients with radiologically resectable peripancreatic tumours. In every instance, the procedure was undertaken immediately before a planned curative resection. In 108 patients in whom a completed extended laparoscopy was performed, 67 were judged to have resectable disease, and 61 of these underwent resection of the cancer (91%). Extended laparoscopy had a failure rate of 9% (missed hepatic metastases in five patients and portal venous encasement in one patient). Overt unresectable disease was identified in 38% (41 patients). The data from this study indicate that extended laparoscopy for pancreatic cancer carries:

- A positive predictive value of 100%.
- A negative predictive value of 91%.
- An accuracy of 94%.

The value of hepatic contact ultrasonography (open and laparoscopic) in the detection of secondary deposits has been documented by several studies including one prospective blinded comparison with preoperative contrast enhanced CT in 77 consecutive patients suffering from colorectal cancer undergoing curative (63 patients) or palliative (14 patients) resections. The patients undergoing curative surgery were randomized to either laparoscopic (34 patients) or conventional open surgery (29 patients) resection. All contact ultrasonographic studies (open and laparoscopic) were performed by two radiologists who were blinded to the CT results. Both laparoscopic contact ultrasonography and CT were negative in 56% (43 patients) and 37 had metastatic disease detected by the three modalities (preop CT, laparoscopic contact ultrasonography, open contact ultrasonography with bimanual palpation). Both open intraoperative contact ultrasonography with bimanual liver palpation and laparoscopic ultrasonography identified hepatic deposits in two patients with negative preoperative contrast-enhanced CT.

## Hepatic resections Approaches

Both the total laparoscopic and the HALS approach can be used although the author's preference is increasingly for the latter except for nonanatomical resections of small nodules for several reasons. In the first instance, the vast majority are anatomical resections (>95%) carried out for metastatic deposits or primary hepatocellular carcinomas. Secondly, the internal hand is the best retractor especially of the right liver during exposure of the retro hepatic vena cava and clipping of the small direct hepatic veins. Finally the HALS approach expedites the operation and provides a most effective safeguard for the arrest of major haemorrhage that may be encountered during the operation. A 7.0 cm incision is necessary for the insertion of the hand access device, such as the Omniport used by the author. This may be midline (better for operations on the left lobe) or right transverse (more suitable for resections on the right liver). It is important that the optical port (right or left) is placed such that it is well clear of the internal hand.

## Assisting hand in HALS

In most instances the surgeon uses his nondominant hand as the assisting internal hand but there is no reason why he should not reverse the situation such that his dominant hand is inside the peritoneal cavity. In a recent experimental study comparing performance between dominant hand of the surgeon inside *vs* outside, better task performance (quality of suturing) and task efficiency (shorter execution time) was observed when the surgeon used the dominant hand inside. In practice the surgeon often has to change hands during HALS hepatic and pancreatic surgery as he moves from one side of the table to the other. This is one of the advantages of operating with the patient in the supine as opposed to the French position (surgeon in between the abducted lower limbs of the patient).

The author finds it useful during certain phases of the operation to have the assistant on the opposite side of the operating table and to insert his right hand internally for retraction. This enables the surgeon to use a two-hand dissection with extracorporeal instruments (more convenient for certain tasks) and gives the surgeon a rest from the lordotic position of the spine imposed by the internal hand being a lower plane that the external one. The size and type of ports selected will depend on the instrumentation and type of dissection used (curved coaxial instruments, ultrasonic dissection, cutting LigaSure Atlas, TissueLink, etc.) Probably, the best compromise for a high-handed surgeon is an 11.0 mm right operating port (to enable use of ultrasonic dissection or LigaSure) and a left 5.5 mm flexible port.

## Selection of patients for the HALS approach and safety precautions during the operations

This is an important consideration that impacts directly on patient outcome. Both laparoscopic and HALS approach for resection of malignant primary and secondary hepatic tumours require for safe execution and technique that ensures an oncologically appropriate resection:

- An adequate residual operating space in the supracolic subdiaphragmatic and subhepatic compartments.
- The resected specimen can be removed *intact* though a protected 7.0 cm mini-laparotomy.

In practice this limits the maximal size of tumour-containing liver resection specimen to 10-cm diameter, i.e. 8.0 cm maximal tumour size. This sensible restriction ensures that the patient obtains all the benefits of the endoscopic approach without compromising cure rate or enhancing the risk of dissemination and loco-regional recurrence.

**Figure 1:**
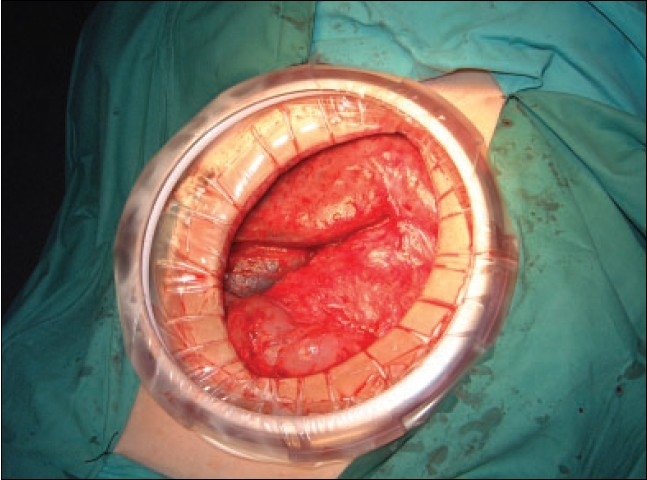
Ecotract disposable wound retractor and protector (Advanced Surgical Inc)

With this restriction all hepatic resections can be carried out by the HALS approach: hepatectomy (right and left, pluri-segmentectomies and bi-/uni-segmentectomies). It is important that the actual hepatic transection by whatever means is performed without positive pressure CO_2_ capnoperitoneum to avoid the risk of CO_2_ embolism through the main hepatic veins. Hence once inflow devascularization of the intended resection is achieved, the CO_2_ insufflation is stopped and in the case of HALS, the hand access device is removed and replaced by a disposable retractor, which also acts as a wound protector [Figure 1] for the actual hepatic resection.

## Component tasks in laparoscopic/HALS hepatic resections

These component tasks cover all the surgical technical aspects of the various hepatic resections: hepatectomy (right or left), pluri-segmentectomies, segmentectomies (right and left).

## Contact ultrasound localization and mapping of the intended resection

**Figure 2:**
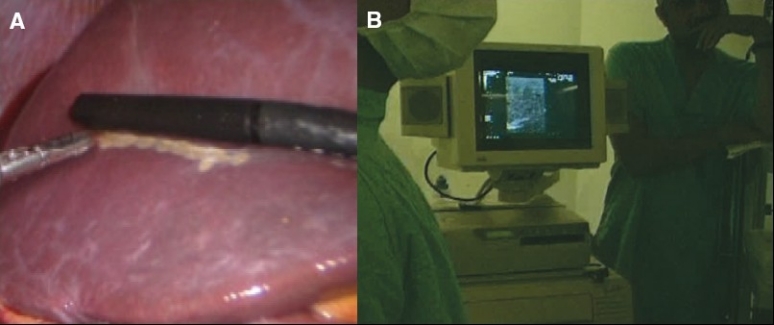
Localisation and surface mapping by soft coagulation of an intraparenchymal lesion

Contact ultrasound is indispensable for laparoscopic and HALS hepatic resections. The precise localization and extent of the lesion especially when this is intra-hepatic can only be determined by contact ultrasound scanning, the findings of which determine the extent of resection (segments) required [Figure 2A, B]. In the case of deep intraparenchymal lesions, the precise location is marked on the surface of the liver by electrocoagulation along the ultrasound probe in two directions. The argon spray plasma coagulation should no be used for this purpose as it may damage the ultrasound probe.

**Figure 3:**
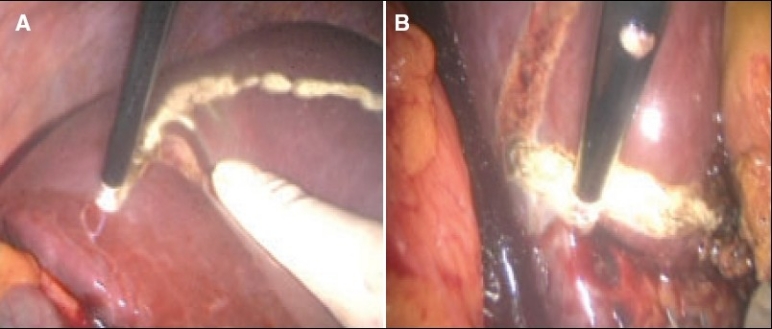
Marking the limits of the hepatic resection with Argon plasma coagulation

In contrast, the mapping of the outlines of the resection is best carried out by the argon plasma spray coagulation. The index finger of the internal assisting hand [Figure 3A] is placed over the centre of the lesion and used to depress the liver whilst the limits of the pluri-segmentectomy (in this case) are outlined on the anterosuperior surface of the liver [Figure 3A, B]. Thereafter the internal hand is used to expose the inferior surface of the liver to complete the marking of the intended hepatic resection.

## Division of falciform ligament, exposure of the suprahepatic vena cava and of the main hepatic veins

**Figure 4:**
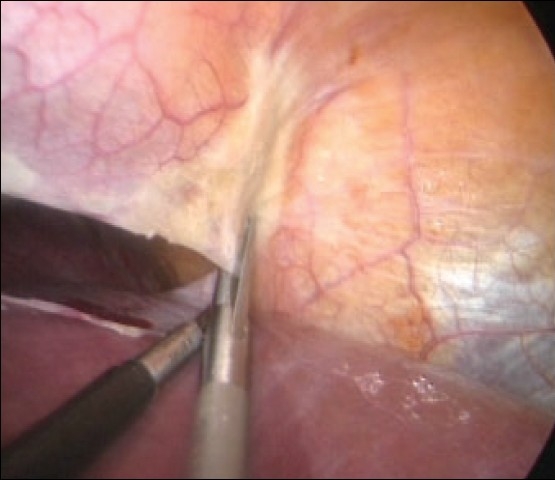
Division of the falciform ligament close to the liver

This component task is obviously needed for major right and left resections. The division of the falciform ligament close to the liver substance is best carried out with a combination of scissors and electrocoagulation and is greatly facilitated by the used of curved coaxial instruments [Figure 4]. The round ligament (Ligamentum Teres) is best left undivided except in patients undergoing skeletonization for right extended hepatectomy.

## Exposure of suprahepatic inferior vena cava and main hepatic veins

**Figure 5:**
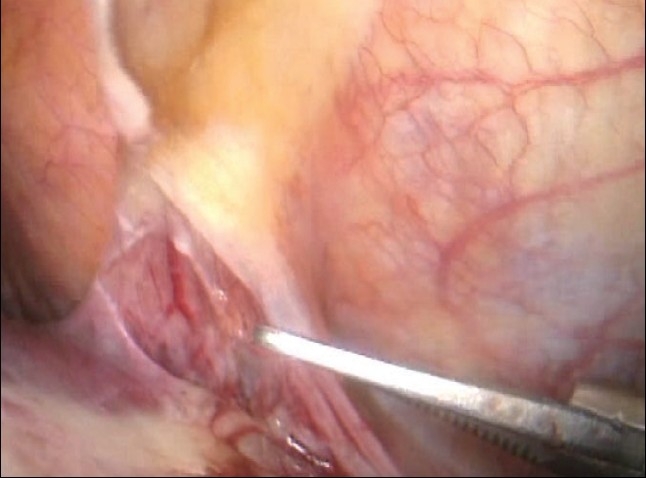
Division of the right and left leaves of the falciform ligament at the back of the liver exposes the upper end of the retrohepatic space containing the inferior vena cava and the main hepatic veins

This is only required for major hepatectomies. The two leaves of the falciform ligament separate posteriorly to envelop the suprahepatic inferior vena cava and the three main hepatic veins. The right leaf becomes the upper leaf of the right (coronary) ligament of the liver and the left leaf, the upper layer of the left (triangular) ligament. These are divided after soft coagulation with the curved coaxial scissors [Figure 5]. Alternatively, ultrasonic shears may be used for this purpose, but this is more difficult as this energized device is straight.

**Figure 6:**
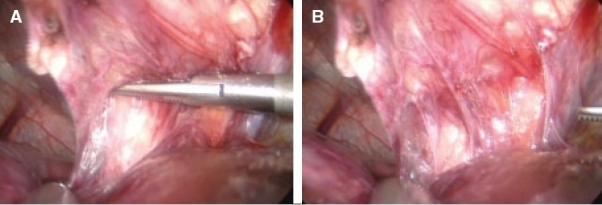
Exposure of the suprahepatic inferior vena cava (white) and the right hepatic vein (tip of scissors) in the groove between the right hepatic vein in front and the inferior vena cava behind

The peritoneal division is extended in both directions to open up the retrohepatic caval space, which consists of relatively avascular loose fibroareolar tissue, and hence most of the dissection is best carried out by the curve coaxial scissors with soft electrocoagulation when needed. The upper end of the caval canal is opened further with a combination of blunt (closed blades) and sharp scissor dissection to divide fibrous bands. As the scissors dissection proceeds, about 1.5 cm of the inferior vena cava, which appears white [Figure 6A, B] and the origin of the right hepatic vein are exposed. Further exposure of the right and middle hepatic veins is achieved form beneath the liver and from the right side (required fro a right hepatectomy). The left hepatic vein is very easily exposed from the left side above the liver.

## Exposure of infrahepatic inferior vena cava and clipping/division of the posterior minor hepatic veins

**Figure 7:**
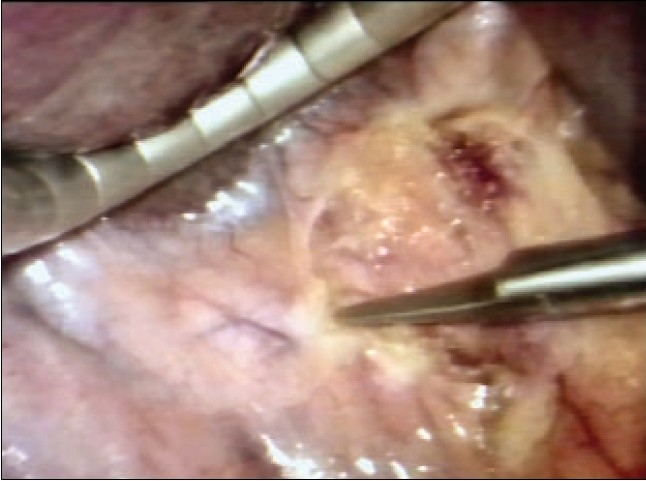
Division of the stretched peritoneum close to the liver to expose the retrohepatic inferior vena cava below and behind the liver. Excess fat is present in this obese patient but this is unusual

This procedure is only necessary for the skeletonization of the right liver necessary for a right hepatectomy. It commences by retraction of the inferior surface of the right lobe of the liver with an atraumatic (flexible ring or fan) retractor to put the peritoneum sweeping up from the right kidney to the liver on the stretch. This peritoneum is divided with the curved coaxial scissors and soft electrocoagulation over a wide front and close to the liver edge. There is usually little fat underneath the peritoneum except in obese individuals [Figure 7].

At this stage, the retractor is replaced by the internal assisting hand, which gently lifts the infero-posterior aspect of the liver upwards to expose the areolar tissue plane covering the vena cava and the minor retrohepatic veins which vary in number from 3 to 5. The infero-posterior aspect of the liver is lifted gently but progressively with the back of the fingers of the internal assisting hand to expose the vena cava behind the liver. As minor hepatic veins are encountered draining into the inferior vena cava, they are skeletonized by the curved coaxial scissors and then clipped before they are divided. The mobilization continues upwards until the right and middle hepatic vein (often one trunk at the origin) is reached.

## Opening the cave of Retius

**Figure 8:**
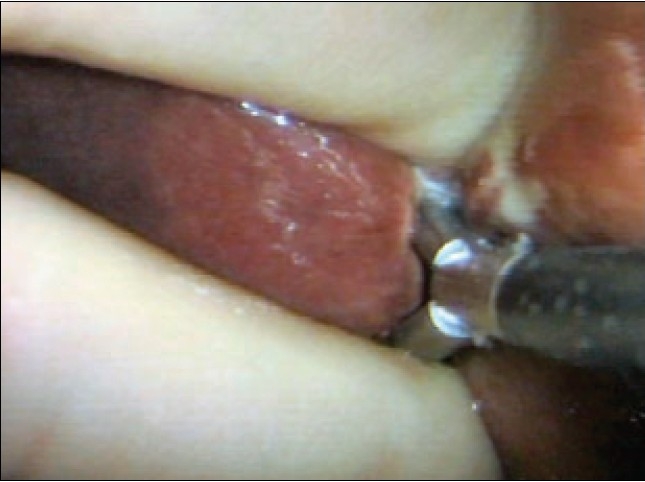
Crush coagulation of the bridge of liver tissue overlying the umbilical fissure of the liver and the round ligament.

**Figure 9:**
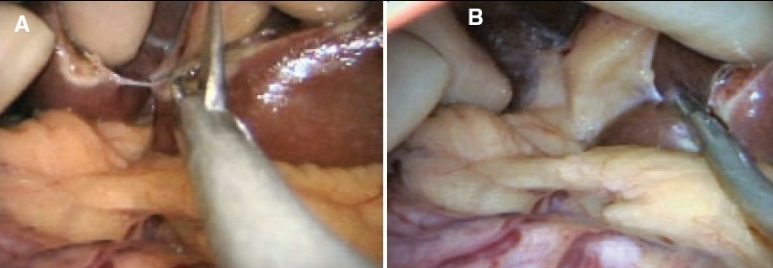
The coagulated and crushed bridge connecting segment III of the left with anterior quadrate lobe (segment IVa) on the right is divided by the curved coaxial scissors to open the cave of Retius and expose the terminal segment of the round ligament.

This procedure is common to both right and left resections. The cave of Retius refers to the umbilical fissure bridged by variable amount of liver tissue anteriorly, which overlies the ligamentum teres (round ligament) containing the obliterated umbilical vein on its way to join the left branch of the portal vein at the bottom of the pit. The bridge of liver tissue is crushed and coagulated by an insulated grasping forceps [Figure 8], after which it is divided thus separating segment III on the left side from the quadrate lobe (anterior segment, IVa) opening up the cave of Retius and exposing the terminal segment of the round ligament [Figure 9A, B].

## Hilar dissection

**Figure 10:**
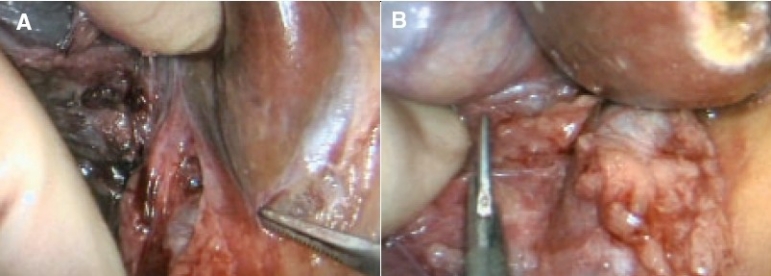
Start of hilar dissection to bring down the hilar plate

The dissection of the hilum commences by division of the peritoneum along the margin of the hepatic hilum [Figure 10A, B] to expose the common hepatic duct and its bifurcation and the right and left branches of the common hepatic artery. Further dissection to bring down the hilar plate and skeletonize the right and left hepatic ducts, the two branches of the common hepatic artery and, more posteriorly, the two branches of the portal vein is needed only for right and left hepatectomy.

## Detachment of the gallbladder

**Figure 11:**
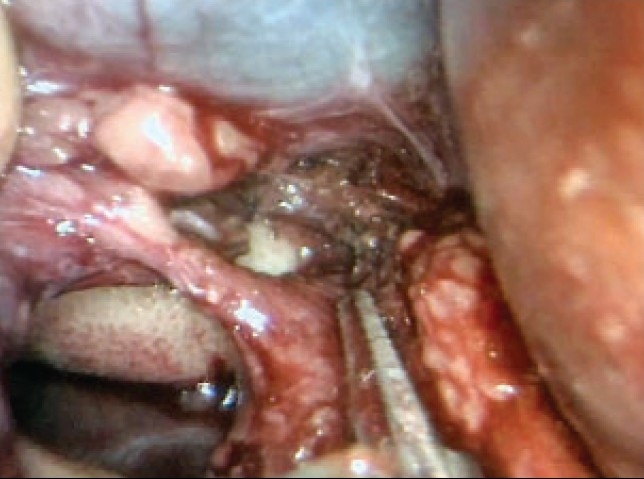
Dissection of the cystic duct and artery during segmentectomy IVa + V

**Figure 12:**
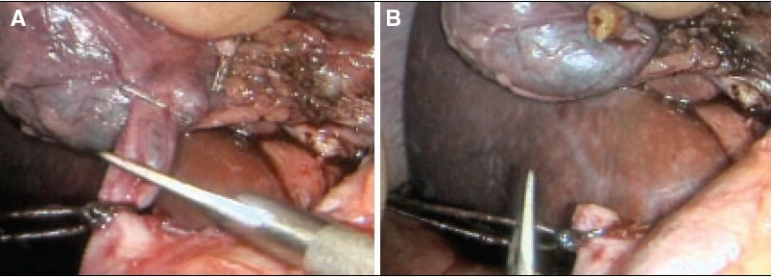
The cystic duct is tied medially with an absorbable suture and clipped laterally before it is cut

Detachment of the gallbladder so that it is removed en block with the hepatic substance constitutes an integral part of right hepatectomy and segmentectomy involving segments IVa and V. The dissection of the cystic duct and artery [Figure 11] is followed by ligature of the medial end of the cystic duct and clipping of its lateral end before it is divided [Figure 12A, B].

**Figure 13:**
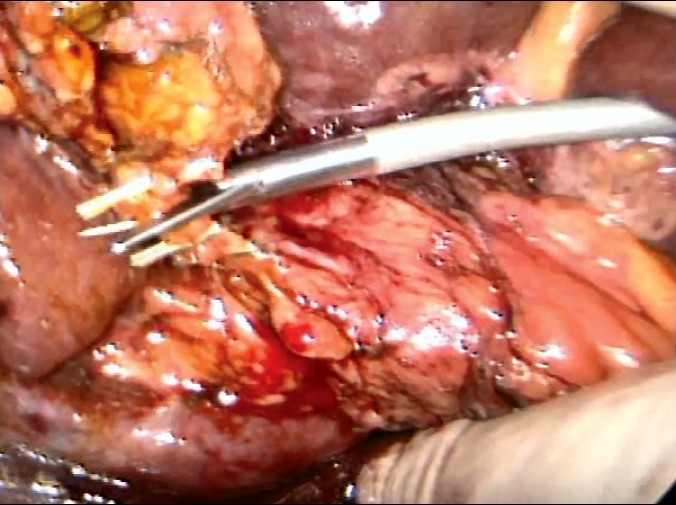
Double clipping and division of the cystic artery

The cystic artery is simply doubly clipped at either end before it is divided with scissors [Figure 13].

## Inflow occlusion prior to hepatic resection

Temporary inflow occlusion of the vascular supply to the liver is necessary for major hepatic resections and also to reduce the “heat-sink effect” of the substantial blood flow through the liver during *in situ* ablation by cryotherapy or radiofrequency thermal ablation. Several varieties of clamps are available for this purpose but the most suited are the parallel occlusion clamps (Storz Tuttlingen), which are introduced through 5.5 mm ports by means of an applicator, which is used to deploy – engage and disengage remove the clamps. Thus when the clamp is in use it does not occupy a port, which can thus be used for dissection. The application of these parallel occlusion clamps is very easy particularly with the HALS approach and minimal dissection is required. The surgeon just makes a small window through an avascular area of lesser omentum just proximal to the hepatoduodenal ligament enveloping the bile duct, hepatic arteries and portal vein.

**Figure 14:**
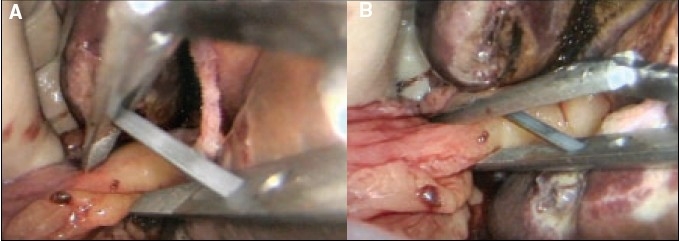
Application of parallel occlusion clamp (through the window in the lesser omentum) to the hepato-duodenal ligament for inflow vascular occlusion to the liver

The parallel occlusion clamp is introduced from the right by means of the dedicated applicator. The jaws are opened as the hepato-duodenal ligament is reached [Figure 14A, B] and applied across the full width of the hepato-duodenal ligament and then released to occlude the bile duct, portal vein and hepatic arteries. It is extremely important that the period of inflow vascular occlusion to the liver does not exceed 30 min at any one-time period.

For removal of the clamp, the introducer is inserted through the port and used to engage the clamp, which is then opened and removed the same port by the introducer.

## Transection of the hepatic parenchyma

The transection of the hepatic parenchyma for all the major resections (hepatectomies, pluri-segmentectomies) should be carried in the absence of a positive-pressure capnoperitoneum. In HALS, this translates to replacement of the hand access device with a disposable retractor that also acts as a wound protector preventing its contamination by malignant cells during the hepatic resection and removal of the specimen [Figure 1]. The hepatic resection must also be carried out with a low patient CVP (around zero), produced by a head-up tilt and appropriate vasodilator medication by the anaesthetist.

The hepatic artery to the resection area is best secured by clips or ligatures in the liver substance rather than extra-hepatically. The vascular stapling or ligature and division of the main hepatic veins draining the liver during hepatectomy (right or left) are carried out at the end of the parenchymal transection.

**Figure 15:**
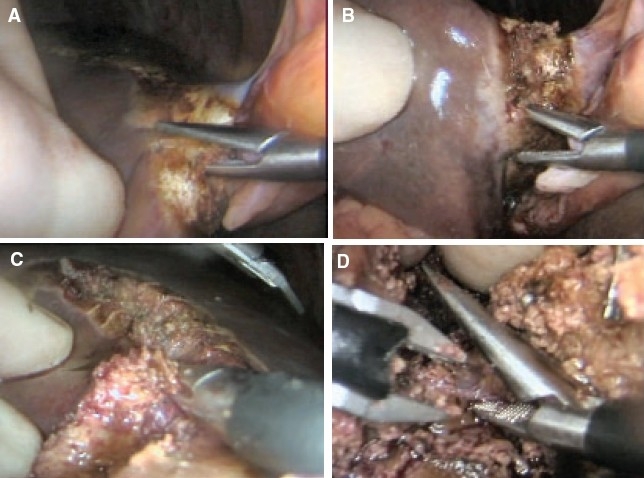
Transection of the liver parenchyma by the laparoscopic crushing forceps technique

The actual technique of liver resection varies from simple finger/forceps fracture with individual clipping/ligature of bile ductules to used of energized systems (Ultrasonic dissection, TissueLink, LigaSure-Atlas). Especially with the HALS approach, the author's preference is for the traditional liver fracture technique. The liver parenchymal surface is first coagulated [Figure 15A] and then crushed using a long-jawed crushing laparoscopic forceps to fracture the liver parenchyma [Figure 15B, C] exposing sizeable vessels and ducts [Figure 15D].

**Figure 16:**
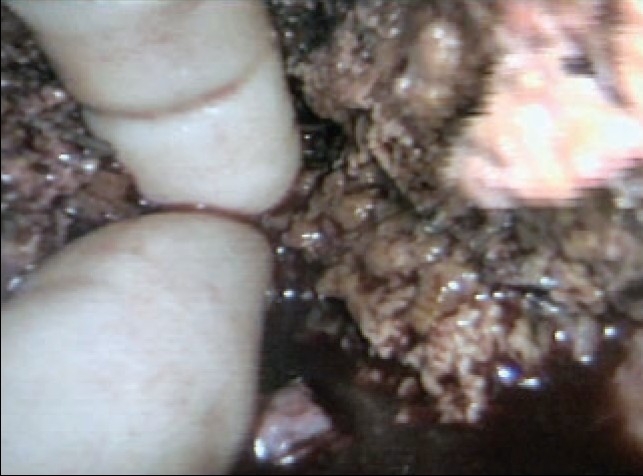
Palpation of a residual bridge following crushing. In this instance a bifurcating interlobar hepatic vein was identified

All sizeable blood vessels and bile ducts are clipped before being cut [Figure 15D]. As the cleft deepens, bands of liver tissue, which are not severed are presumed to contain large vessels which may be obscured by adherent layer of liver parenchyma. In this situation, palpation of the bridge between the index finger and thumb of the assisting hand will identify the nature of the structure [Figure 16].

**Figure 17:**
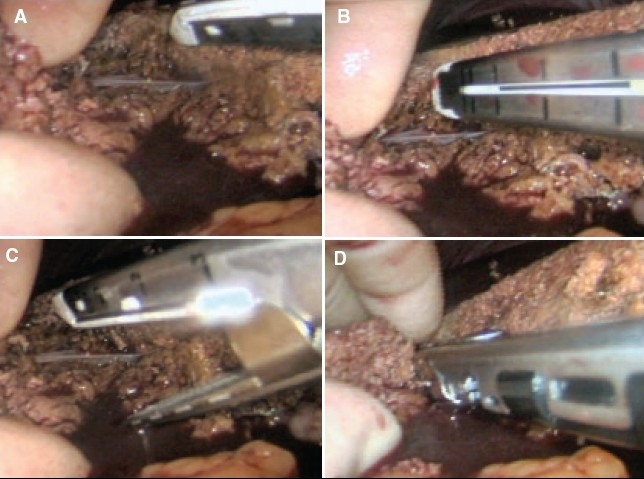
Stapling of large interlobar hepatic vein with the endolinear cutting stapler. The new generation of deflecting (roticulator type) staplers are ideal for this procedure

All sizeable veins are best staple transected using an endolinear cutting stapler mounted with 35 mm vascular cartridge introduced through the mini-laparotomy wound [Figure 17A–D].

**Figure 18:**
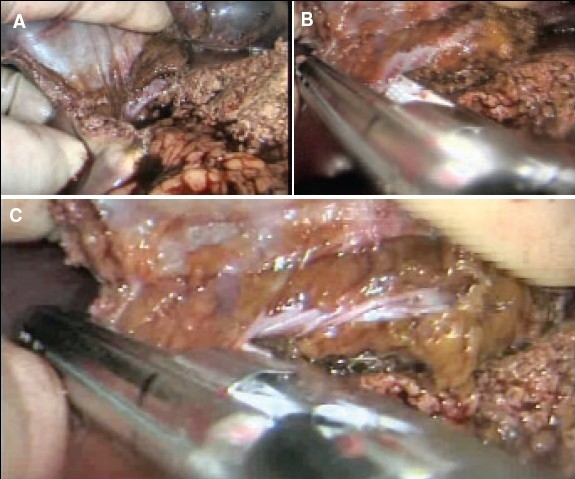
Detachment of the resected area from the residual liver substance by cross stapling through the hepatic parenchyma. This is safe provided the connecting bridge is not thicker than 1.0cm

**Figure 19:**
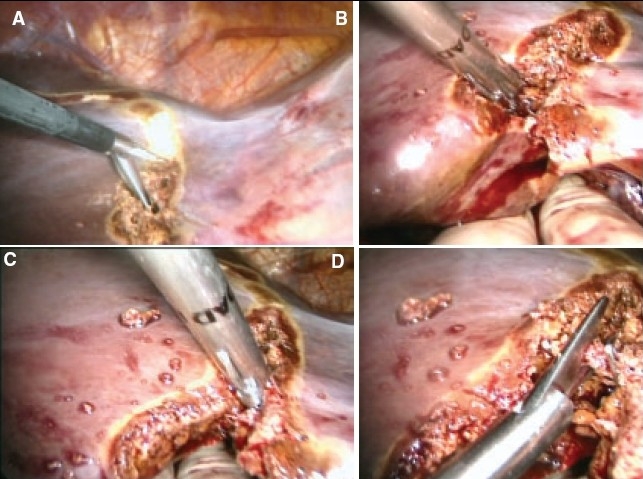
Separation of hepatic parenchyma using fracture technique with clipping of vessels and bile ductules during right hepatectomy

In the case of pluri-segmentectomy, after the segment has been separated on three sides, it often remains attached to the liver by bridge of liver tissue [Figure18A]. If this connection is no thicker than 1.0 cm, it is simply staple transected by the application of the endolinear cutting stapler [Figure 18B, C] to detach completely the area from the liver. Essentially the same fracture technique of transection of the liver parenchyma is used for right and left hepatectomy [Figure 19A–D].

The coaxial curved duckbill forceps is very useful for getting round large veins in the depths of the transection cleft prior to clipping or ligature. It may be difficult because of insufficient space to use staplers in this situation especially as the diaphragm is approached.

When the resection is completed, the specimen is removed through the open mini-laparotomy wound. The final stage consists in securing complete haemostasis.

## Haemostasis of the cut liver surface

**Figure 20:**
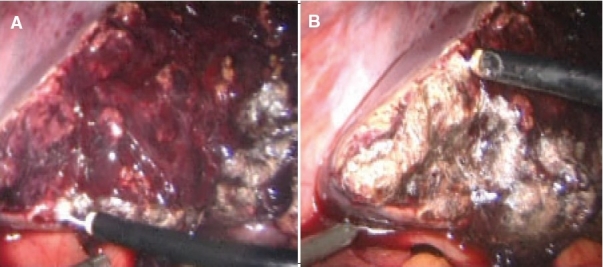
Argon plasma spray coagulation of the cut liver substance, starting at the bottom and working upwards

There should only be a minor oozing from the cut liver substance if the technique of hepatic transection has been performed correctly and in the presence of a low patient CVP. Complete haemostasis is achieved by argon plasma coagulation followed by the application of fibrin glue or other synthetic sealant. There is no risk attached to any long use of the argon plasma spray coagulation as the wound is open and there is no positive pressure. The technique of argon plasma coagulation of the liver substance is important. In the first instance the tip of the probe should be held a few mm from the liver surface and must not touch the liver such that the plasma jet is clearly seen [Figure 20A]. The plasma jet has a tendency to move away from dried up areas to wet (oozy) areas. Thus care must be taken to avoid the plasma from straying to important structures near to the liver. The plasma spray coagulation should start ate the bottom of the transected liver substance and then proceed upwards [Figure 20B].

**Figure 21:**
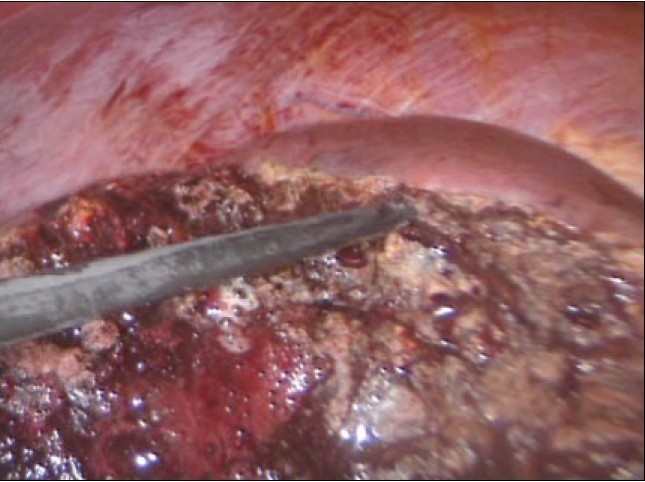
The coagulated liver surface is covered with fibrin glue

Once the entire surface is coagulated, it is covered by fibrin glue or an alternative synthetic sealant [Figure 21].

## Insertion of silicon drains

**Figure 22:**
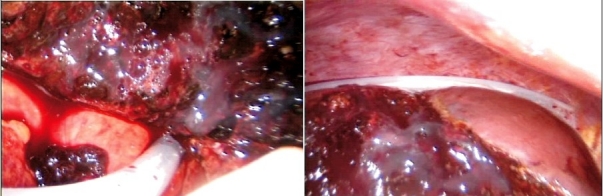
Insertion of drains above and below the liver is essential

This is the final step of the operation before the retractor is removed and the wound closed using mass closure with monofilament polydioxanone. It is advisable to inserted two large silicon drains one above and the other below the liver [Figure 22A, B]. These must be sutured to the abdominal wall to prevent accidental dislodgement after the operation. Effective drainage is crucial to prevent postoperative biloma.

## Postoperative management

In the first instance, it is important o stress that these patients should be nursed postoperatively in a hepato-biliary unit with immediate access to high dependency and intensive care if needed.

The management is the same as after open surgery with daily monitoring of the liver functions tests, haematology and blood urea nitrogen and serum electrolytes. Opiate medication and sedation are avoided in patients with compromised liver function, e.g. Childs C disease. An ultrasound scan should be carried out in all patients after hepatic resection at 48 h. This is necessary to identify early fluid collections (most usually bile), which if found are monitored by serial ultrasound studies and aspirated or drained percutaneously under radiological control if persistent. Fluids are started the next day and most patients should be on oral diet 24 h after the intervention. The abdominal drains are removed on the third day if dry or producing only a small amount of serous fluid. The stay in hospital depends on the state of the liver function and the extent of the resection. Undoubtedly, however, these patients recover more quickly in terms of hospital stay and period of short-term disability than after open hepatic resections.

## CONCLUSIONS

With the right technique, necessary expertise and appropriate technology, laparoscopic and especially HALS hepatic resections for tumours (primary and secondary carcinomas) can be carried out safely. The data from the published reports to date indicate benefits over the open approach and these include reduced blood loss and lower postoperative morbidity. In the author's opinion all major resections are carried more expeditiously with increased safety and less stress to the surgeon and the operating team by the HALS approach.
